# Comparison of Intracranial Pressure Measurements Before and After Hypertonic Saline or Mannitol Treatment in Children With Severe Traumatic Brain Injury

**DOI:** 10.1001/jamanetworkopen.2022.0891

**Published:** 2022-03-10

**Authors:** Patrick M. Kochanek, P. David Adelson, Bedda L. Rosario, James Hutchison, Nikki Miller Ferguson, Peter Ferrazzano, Nicole O’Brien, John Beca, Ajit Sarnaik, Kerri LaRovere, Tellen D. Bennett, Akash Deep, Deepak Gupta, F. Anthony Willyerd, Shiyao Gao, Stephen R. Wisniewski, Michael J. Bell

**Affiliations:** 1 Department of Critical Care Medicine, University of Pittsburgh, Pittsburgh, Pennsylvania; 2 Barrow Neurological Institute at Phoenix Children’s Hospital, Phoenix, Arizona; 3 Department of Epidemiology, University of Pittsburgh, Pittsburgh, Pennsylvania; 4 Department of Critical Care Medicine, Toronto Sick Children’s Hospital, Toronto, Ontario, Canada; 5 Department of Pediatrics, Virginia Commonwealth University School of Medicine, Richmond; 6 Department of Pediatrics, University of Wisconsin School of Medicine, Madison; 7 Department of Pediatrics, The Ohio State University School of Medicine, Columbus; 8 Department of Pediatrics, Starship Children’s Hospital, Auckland, New Zealand; 9 Department of Pediatrics, Wayne State University, Detroit, Michigan; 10 Department of Neurology, Boston Children’s Hospital, Boston, Massachusetts; 11 Department of Pediatrics, University of Colorado School of Medicine, Aurora; 12 Department of Pediatrics, King’s College Hospital NHS Foundation Trust, London, UK; 13 Department of Neurosurgery, All India Institute of Medical Sciences, New Delhi; 14 Department of Pediatrics, Children’s National Hospital, Washington, DC

## Abstract

**Importance:**

Hyperosmolar agents are cornerstone therapies for pediatric severe traumatic brain injury. Guideline recommendations for 3% hypertonic saline (HTS) are based on limited numbers of patients, and no study to date has supported a recommendation for mannitol.

**Objectives:**

To characterize current use of hyperosmolar agents in pediatric severe traumatic brain injury and assess whether HTS or mannitol is associated with greater decreases in intracranial pressure (ICP) and/or increases in cerebral perfusion pressure (CPP).

**Design, Setting, and Participants:**

In this comparative effectiveness research study, 1018 children were screened and 18 were excluded; 787 children received some form of hyperosmolar therapy during the ICP-directed phase of care, with 521 receiving a bolus. Three of these children were excluded because they had received only bolus administration of both HTS and mannitol in the same hour, leaving 518 children (at 44 clinical sites in 8 countries) for analysis. The study was conducted from February 1, 2014, to September 31, 2017, with follow-up for 1 week after injury. Final analysis was performed July 20, 2021.

**Interventions:**

Boluses of HTS and mannitol were administered.

**Main Outcomes and Measures:**

Data on ICP and CPP were collected before and after medication administration. Statistical methods included linear mixed models and corrections for potential confounding variables to compare the 2 treatments.

**Results:**

A total of 518 children (mean [SD] age, 7.6 [5.4] years; 336 [64.9%] male; 274 [52.9%] White) were included. Participants’ mean (SD) Glasgow Coma Scale score was 5.2 (1.8). Bolus HTS was observed to decrease ICP and increase CPP (mean [SD] ICP, 1.03 [6.77] mm Hg; *P* < .001; mean [SD] CPP, 1.25 [12.47] mm Hg; *P* < .001), whereas mannitol was observed to increase CPP (mean [SD] CPP, 1.20 [11.43] mm Hg; *P* = .009). In the primary outcome, HTS was associated with a greater reduction in ICP compared with mannitol (unadjusted β, −0.85; 95% CI, −1.53 to −0.19), but no association was seen after adjustments (adjusted β, −0.53; 95% CI, −1.32 to 0.25; *P* = .18). No differences in CPP were observed. When ICP was greater than 20 mm Hg, greater than 25 mm Hg, or greater than 30 mm Hg, HTS outperformed mannitol for each threshold in observed ICP reduction (>20 mm Hg: unadjusted β, −2.51; 95% CI, −3.86 to −1.15, *P* < .001; >25 mm Hg: unadjusted β, −3.88; 95% CI, −5.69 to −2.06, *P* < .001; >30 mm Hg: unadjusted β, −4.07; 95% CI, −6.35 to −1.79, *P* < .001), with results remaining significant for ICP greater than 25 mm Hg in adjusted analysis.

**Conclusions and Relevance:**

In this comparative effectiveness research study, bolus HTS was associated with lower ICP and higher CPP, whereas mannitol was associated only with higher CPP. After adjustment for confounders, both therapies showed no association with ICP and CPP. During ICP crises, HTS was associated with better performance than mannitol.

## Key Points

**Question:**

How commonly are hyperosmolar therapies used in the management of severe traumatic brain injury in children, and which agent—mannitol or hypertonic saline solution—is associated with greater decreases in intracranial pressure (ICP) and/or increases in cerebral perfusion pressure (CPP)?

**Findings:**

In this comparative effectiveness research study of 1000 consecutive children with traumatic brain injury, more than 77% of the study population received hyperosmolar therapies during the ICP treatment phase of their care, and almost 2500 bolus administrations of 3% hypertonic saline and mannitol were analyzed. Hypertonic saline was associated with decreased ICP, whereas hypertonic saline and mannitol had similar observed effectiveness for CPP.

**Meaning:**

This study of children with severe traumatic brain injury found that bolus administration of hypertonic saline was associated with superior ICP and CPP outcomes.

## Teaser

This comparative effectiveness research study of children with traumatic brain injury (TBI) compares intracranial pressure and cerebral perfusion pressure outcomes of bolus administration of hypertonic saline and mannitol.

## Introduction

Severe traumatic brain injury (sTBI) in children is an enormous worldwide problem, leading to more than 2500 US deaths in 2014.^1^ Traumatic brain injury contributes to lifelong disability in survivors. On the basis of recent data addressing the global burden of neurologic disorders, TBI was in the top 10 causes of years lost to disability throughout infancy, childhood, and adolescence.^2^

Hyperosmolar therapy has been a cornerstone in the management of pediatric sTBI.^3^ An early study^4^ considered the use of mannitol or glycerol, but support emerged specifically for the utility of 3% hypertonic saline (HTS). As a bolus or continuous infusion, HTS has been used to reduce intracranial pressure (ICP) and/or improve cerebral perfusion pressure (CPP).^5,6,7,8^ Indeed, the 3% concentration is the only therapy that generated a supportive level 2 treatment recommendation for use in the Brain Trauma Foundation Guidelines for the Management of Severe Pediatric TBI.^9,10^ Because these recommendations were based on studies of only 121^9^ and 169^10^ patients, the evidence was considered relatively weak. Despite mannitol being in clinical practice for decades, to our knowledge, no study evaluating mannitol has qualified for inclusion in the guideline. Given the paucity of high-quality evidence, the association between ICP and mortality,^11,12^ and the importance of hyperosmolar therapy in the current guidelines, we conducted a comparative effectiveness research study to characterize current use of hyperosmolar agents in pediatric sTBI and tested hypotheses regarding bolus administration of HTS and mannitol on ICP and CPP.

## Methods

### Study Design

This observational comparative effectiveness research analysis uses data from the Approaches and Decisions for Acute Pediatric TBI Trial (ADAPT), an observational cohort study funded by a cooperative agreement with the National Institute of Neurological Disorders and Stroke. ADAPT included recruitment sites in the US, UK, Spain, the Netherlands, India, South Africa, Australia, and New Zealand. Centers obtained institutional human research review board approval to enroll children, and the University of Pittsburgh received institutional review board approval. All sites were permitted to perform data collection, including patient characteristics and standard therapies administered, before informed consent. Written informed consent was provided for long-term outcomes that were not part of this study. Informed consent was not required for data collection at all clinical sites. Thus, this cohort represents consecutive children who met the inclusion and exclusion criteria at the sites. This study followed the Strengthening the Reporting of Observational Studies in Epidemiology (STROBE) reporting guideline.

Inclusion criteria were age from birth to 18 years at the time of injury, diagnosis of TBI, ICP monitor used as part of the standard care, and Glasgow Coma Scale (GCS) score of 8 or lower at the time of monitor placement. Exclusion criteria were pregnancy and ICP monitor placement at an institution other than the clinical site. A total of 1018 children were screened, and 18 were excluded for having an ICP monitor placed outside a study site for the overall study (Figure). A total of 787 children received hyperosmolar therapy during the ICP-directed phase of care, with 521 receiving a bolus. Three of these children were excluded because they had only bolus administrations of HTS and mannitol in the same hour during the study period, leaving 518 children (at 44 clinical sites in 8 countries) for analysis (eTable 3 in Supplement 1).

**Figure.**
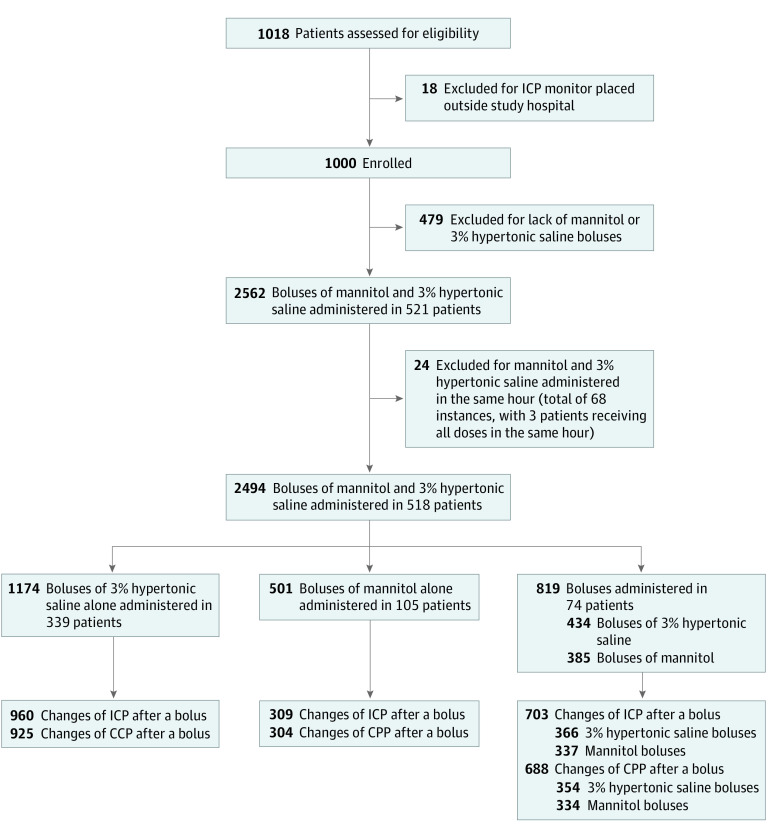
Patient Selection Flowchart CPP indicates cerebral perfusion pressure; ICP, intracranial pressure.

Several phases of care were identified. The prehospital phase (time of injury until arrival at the study site), resuscitation phase (time from when the participant arrived at the study site until ICP monitor placement), and the ICP-directed therapy phase (up to 7 days after ICP monitor placement or until the ICP monitor removal) were defined.^13^ For this analysis, only the ICP-directed therapy phase was used so that ICP and CPP effectiveness could be compared. Demographic characteristics, injury details, prehospital and resuscitation events, medications, and other variables were collected, consistent with the National Institute of Neurological Disorders and Stroke TBI Common Data Elements (eTable 1 in Supplement 1).^14^

A bolus administration was defined as the administration of HTS or mannitol in 1 hour of data collection, whereas no other hyperosmolar agents were administered in the hour before or after the index administration. Thus, instances when hyperosmolar therapies were administered in consecutive hours were excluded, and continuous infusions of HTS were not considered. Concentrations of all HTS administered in the study and concentrations of all HTS boluses are described in eTables 2 and 3 in Supplement 1. Only 3% HTS boluses (1642 of 2174 [75.5%] of all boluses administered) were included in this analysis. The HTS and mannitol doses were indexed to body weight (milliliters per kilogram for HTS and grams per kilogram for mannitol). Comparison of ICP (or CPP) in the hour before with the hour after bolus administration was calculated. The study was conducted from February 1, 2014, to September 31, 2017, with follow-up for 1 week after injury. Final analysis was performed July 20, 2021.

### Statistical Analysis

Baseline characteristics were summarized and reported as mean (SD) or numbers (percentages), as appropriate. A linear mixed model (LMM) to compare the changes in ICP (or CPP) associated with HTS and mannitol with the hyperosmolar treatment indicator as the independent variable was developed. Because of the large number of potential confounders, we applied variable selection for LMMs by L1-penalized estimation based on the R software package glmmLasso.^15^ This generalized LMM with Lasso approach is an extension of Lasso, which allows for the inclusion of random effects. Potential confounding characteristics (dose, study day, and all baseline characteristics of study participants) that were selected a priori and entered into the model as independent variables of interest and changes in ICP (or CPP) were considered outcomes of interest. For each model, we included patient level as a random intercept, and a treatment indicator variable was forced to be included in the model. Because the analysis only allows complete case scenario, observations with incomplete data were excluded (remaining n = 1609 in the ICP analysis and n = 1562 in the CPP analysis). To optimize the tuning parameter λ, we applied bayesian information criterion–based selection. After potential confounding data variable selection, we refitted the LMMs and estimated the adjusted effect sizes of HTS vs mannitol on the changes in ICP (or CPP) by including the independent variables selected via glmmLasso.

Subgroup analyses were conducted to compare the changes in ICP (or CPP) associated with HTS and mannitol based on ICP level in the hour before administration (cutoff points of >20, >25, and >30 mm Hg). The same LMMs used in the overall analyses were extended to include an interaction term between the treatment indicator variable and the baseline characteristic. Because the observed outcomes of hyperosmolar therapy observed may be blunted over time,^16^ a sensitivity analysis was performed to compare the outcomes of the first bolus of a hyperosmolar medication for each patient. Unadjusted linear regression models were used to assess the outcomes of the first bolus of HTS and mannitol on the differences of ICP (and CPP). Propensity score analysis was used to reduce confounding or selection bias of observed baseline characteristics. We estimated the propensity scores using the generalized boosted models approach and considered all baseline characteristics as mentioned earlier.^17,18^ We applied propensity score–weighted linear regression to compare the association of HTS and mannitol with the changes of ICP (or CPP). All analyses were 2-sided, and the significance level was set to *P* < .05. Analyses were conducted using SAS software, version 9.4 (SAS Institute Inc) and R software, version 3.6.2 (R Foundation for Statistical Computing).

## Results

A total of 518 children (mean [SD] age, 7.6 [5.4] years; 336 [64.9%] male; 115 [22.2%] Black, 51 [10.0%] Latino, 274 [52.9%] White, 100 [19.3%] other [American Indian or Alaska Native, Asian, Native Hawaiian or Pacific Islander, and >1 race]; 29 [5.6%] unknown or withheld) were studied. Select characteristics of these children are given in Table 1, all baseline characteristics of study participants are summarized in eTable 4 in Supplement 1, and boluses administered at each clinical site are summarized in eTable 5 in Supplement 1. In our study, 30.3% of patients received a decompressive craniectomy and 34.4% received cerebrospinal fluid diversion.

**Table 1.** Selected Baseline Characteristics of Patients Included in the Analysis^a^

Characteristic	Patients included in the current analysis of bolus therapy (N = 518)	Patients who received other hyperosmolar therapies and were not included in the current study (n = 296)	Patients without any hyperosmolar therapies (n = 185)
Age, mean (SD), y	7.6 (5.4)	7.9 (5.5)	7.0 (5.2)
Sex			
Female	182 (35.1)	101 (34.1)	72 (38.9)
Male	336 (64.9)	195 (65.9)	113 (61.1)
Primary race			
Black	115 (22.2)	53 (17.9)	45 (24.3)
White	274 (52.9)	182 (61.5)	104 (56.2)
Other^b^	100 (19.3)	17 (5.7)	27 (14.6)
Unknown or withheld	29 (5.6)	44 (14.9)	9 (4.9)
Latino			
Not available	188 (36.3)	115 (38.9)	73 (39.5)
No	272 (52.5)	132 (44.6)	91 (49.2)
Yes	51 (9.8)	44 (14.9)	16 (8.6)
Unknown	7 (1.4)	5 (1.7)	5 (2.7)
Cause of injury			
Motor vehicle crash	289 (55.8)	161 (54.4)	106 (57.3)
Fall	103 (19.9)	44 (14.9)	34 (18.4)
Homicide or assault	77 (14.9)	50 (16.9)	20 (10.8)
Other	49 (9.5)	41 (13.8)	25 (13.5)
Type of injury			
Open	51 (9.8)	28 (9.5)	17 (9.2)
Closed	467 (90.2)	268 (90.5)	168 (90.8)
Mechanism of injury			
Acceleration or deceleration	49 (9.6)	29 (10.0)	17 (9.2)
Direct impact or fall	429 (83.8)	245 (84.2)	159 (86.4)
Penetrating	34 (6.6)	17 (5.8)	8 (4.4)
Likelihood injury due to abuse			
No concern	426 (82.2)	239 (80.7)	156 (84.3)
Possible	26 (5.0)	10 (3.4)	11 (6.0)
Probable	34 (6.6)	23 (7.8)	11 (6.0)
Definite	32 (6.2)	24 (8.1)	7 (3.8)
Glasgow Coma Scale total score, mean (SD)	5.2 (1.8)	5.2 (1.8)	5.0 (1.8)
Injury Severity Score, mean (SD)	26.3 (11.6)	27.9 (11.1)	26.7 (12.5)
Cardiac arrest			
No	475 (91.7)	269 (90.9)	174 (94.1)
Yes	43 (8.3)	27 (9.1)	11 (5.9)
Pediatric Risk of Mortality III Score, mean (SD)	17.0 (9.1)	17.6 (9.4)	16.2 (8.3)
Fixed pupils			
Both	117 (22.6)	57 (19.3)	30 (16.2)
Either	51 (9.8)	27 (9.1)	17 (9.2)
Neither	310 (59.8)	194 (65.5)	121 (65.4)
Unable to assess or unknown	40 (7.7)	18 (6.1)	17 (9.2)
Hypertonic saline in resuscitation			
No	326 (62.9)	163 (55.1)	139 (75.1)
Yes	192 (37.1)	133 (44.9)	46 (24.9)
Mannitol in resuscitation			
No	359 (69.3)	219 (74.0)	151 (81.6)7
Yes	159 (30.7)	77 (26.0)	34 (18.4)

^a^ Data are presented as number (percentage) of patients unless otherwise indicated. A total of 814 (518 + 296) patients received hyperosmolar therapy during the intracranial pressure–directed phase of care. Data for hyperosmolar therapy were not available for 1 patient enrolled in the Approaches and Decisions for Acute Pediatric TBI Trial.

^b^ Includes American Indian/Alaska Native, Asian, Native Hawaiian/Pacific Islander, and more than 1 race.

In total, 339 patients were treated with HTS boluses, 105 patients were treated with mannitol boluses, and 74 patients were treated with both HTS and mannitol boluses, with the number of doses administered outlined in the Figure. For ICP, 1326 HTS boluses and 646 mannitol boluses had complete data for analysis, whereas for CPP, 1279 HTS boluses and 638 mannitol boluses had analyzable data. During the ICP-directed phase, 7.4% received only mannitol (with an additional 27.2% receiving both mannitol and HTS). Additional details regarding boluses are available in eTables 6 and 7 and eFigures 1 and 2 in Supplement 1. The mean (SD) bolus doses were 4.78 (2.89) mL/kg for HTS and 0.48 (0.34) g/kg for mannitol, corresponding to 4.91 (2.97) mOsm/kg for HTS and 2.66 (1.86) mOsm/kg for mannitol. Hypertonic saline was given earlier than mannitol (eTable 8 in Supplement 1). Finally, 78.4% of boluses of hyperosmolar therapy were given to patients with ICPs of 20 mm Hg or less recorded in the hour before the dose (eTable 9 in Supplement 1).

The observed decrease in mean (SD) ICP for HTS was 1.03 (6.77) mm Hg (*P* < .001), whereas the observed decrease for mannitol was 0.20 (6.53) mm Hg (*P* = .44). The observed increase in mean (SD) CPP for HTS was 1.25 (12.47) mm Hg (*P* < .001), whereas the observed increase for mannitol was 1.20 (11.43) mm Hg (*P* = .009). In an unadjusted analysis, HTS was associated with a larger observed association with ICP than mannitol (unadjusted β, −0.85; 95% CI, −1.53 to −0.19; *P* = .01). However, after adjustment for dosing, GCS verbal score, sex, cause of injury, likelihood of injury from abuse, likelihood of intentional injury, lower extremity Abbreviated Injury Scale score, and epidural hematoma, the observed associations of the 2 agents were not different (unadjusted β, −0.53; 95% CI, −1.32 to 0.25; *P* = .18). No observed CPP differences were found between HTS and mannitol (unadjusted β, 0.05; 95% CI, −1.10 to 1.20; *P* = .93; adjusted β, −0.71; 95% CI, −2.05 to 0.62; *P* = .30).

We assessed the association of HTS and mannitol with ICP and CPP observed during varying degrees of intracranial hypertension (ICP >20, >25, and >30 mm Hg). For ICP greater than 20 mm Hg, a total of 427 doses of hyperosmolar therapy were analyzable (299 for HTS and 128 for mannitol), and HTS was associated with a greater ICP response than mannitol (ICP decrease: 5.48 [7.94] mm Hg vs 2.91 [9.56] mm Hg) (Table 2). At ICP thresholds of greater than 25 mm Hg and greater than 30 mm Hg, similar reductions in ICP were observed (Table 2). In an unadjusted analysis, HTS was associated with a higher ICP decrease than mannitol at all 3 thresholds (>20 mm Hg: unadjusted β, −2.51; 95% CI, −3.86 to −1.15; *P* < .001; >25 mm Hg: unadjusted β, −3.88; 95% CI, −5.69 to −2.06; *P* < .001; >30 mm Hg: unadjusted β, −4.07; 95% CI, −6.35 to −1.79; *P* < .001) (Table 3). In an adjusted model, HTS was associated with a greater ICP response than mannitol at ICP greater than 25 mm Hg (adjusted β, −2.94; 95% CI, −5.13 to −0.75; *P* = .01) (Table 3). In contrast, HTS and mannitol had no observed association with CPP response in unadjusted or adjusted analyses (>20 mm Hg: unadjusted β, 1.18; 95% CI, −1.32 to 3.69; *P* = .35; adjusted β, 0.06; 95% CI, −3.00 to 2.88; *P* = .97; >25 mm Hg: unadjusted β, −0.16; 95% CI, −1.38 to 1.05; *P* = .23; adjusted β, 0.58; 95% CI, −3.42 to 4.58; *P* = .78; >30 mm Hg: unadjusted β, −0.20; 95% CI, −4.40 to 4.01; *P* = .93; adjusted β, −2.20; 95% CI, −7.35 to 2.94; *P* = .40) (eTable 10 in Supplement 1).

**Table 2.** Change in Intracranial Pressure (ICP) After Administration of 3% Hypertonic Saline and Mannitol Boluses Stratified by the ICP Level Recorded in the Hour Before the Dose

Stratum	Total	3% Hypertonic saline	Mannitol
ICP ≤20 mm Hg, No.	1545	1027	518
Mean (95% CI)	0.33 (0.05 to 0.62)	0.26 (−0.09 to 0.62)	0.47 (0.01 to 0.93)
Median (IQR)	0 (−2 to 2)	0 (−3 to 2)	0 (−2 to 2)
ICP >20 mm Hg	427	299	128
Mean (95% CI)	−4.71 (−5.52 to −3.90)	−5.48 (−6.39 to −4.57)	−2.91 (−4.59 to −1.24)
Median (IQR)	−4 (−9 to 0)	−5 (−10 to 0)	−2 (−7 to 1)
ICP ≤25 mm Hg	1750	1181	569
Mean (95% CI)	−0.13 (−0.40 to 0.14)	−0.28 (−0.62 to 0.06)	0.18 (−0.28 to 0.63)
Median (IQR)	0 (−3 to 2)	0 (−3 to 2)	0 (−2 to 2)
ICP >25 mm Hg	222	145	77
Mean (95% CI)	−5.71 (−7.07 to −4.35)	−7.17 (−8.73 to −5.60)	−2.96 (−5.48 to −0.44)
Median (IQR)	−5 (−12 to 0)	−7 (−12 to −1)	−2 (−8 to 2)
ICP ≤30 mm Hg	1836	1244	592
Mean (95% CI)	−0.45 (−0.73 to −0.17)	−0.66 (−1.01 to −0.31)	−0.01 (−0.48 to 0.45)
Median (IQR)	0 (−3 to 2)	−1 (−4 to 2)	0 (−3 to 2)
ICP >30 mm Hg	136	82	54
Mean (95% CI)	−4.90 (−6.80 to −2.99)	−6.65 (−8.94 to −4.36)	−2.24 (−5.52 to 1.04)
Median (IQR)	−3 (−12 to 1.5)	−6 (−13 to 0)	−1 (−7 to 3)

**Table 3.** Unadjusted and Adjusted Associations of 3% Hypertonic Saline vs Mannitol With the Change of Intracranial Pressure (ICP) After a Bolus Stratified by the ICP Level Recorded in the Hour Before the Dose

Stratum	Unadjusted β (95% CI)	*P* value^a^	Adjusted β (95% CI)^b^	*P* value^a^
ICP, mm Hg				
≤20	−0.35 (−1.09 to 0.38)	.35	−0.42 (−1.29 to 0.44)	.34
>20	−2.51 (−3.86 to −1.15)	<.001	−1.50 (−3.13 to 0.12)	.07
ICP, mm Hg				
≤25	−0.58 (−1.29 to 0.12)	.11	−0.41 (−1.24 to 0.41)	.32
>25	−3.88 (−5.69 to −2.06)	<.001	−2.94 (−5.13 to −0.75)	.01
ICP, mm Hg				
≤30	−0.70 (−1.39 to −0.003)	.05	−0.47 (−1.27 to 0.32)	.24
>30	−4.07 (−6.35 to −1.79)	<.001	−2.77 (−5.60 to 0.05)	.058

^a^ *P* < .05 for interactions for all unadjusted analyses and adjusted analysis when ICP in the hour before was dichotomized at 25 mm Hg.

^b^ Adjusted for dosing, verbal Glasgow Coma Scale score, sex, cause of injury, likelihood of injury being caused by abuse, likelihood of intentional injury, lower-extremity Abbreviated Injury Scale score, and epidural hematoma.

In comparing the response to the first bolus, we found that HTS was associated with a larger decrease in ICP compared with mannitol in an unadjusted model (unadjusted β, −1.99; 95% CI, −3.82 to −0.16; *P* = .03), but no association was seen in adjusted and propensity-weighted models (adjusted β, −2.74; 95% CI, −5.61 to 0.13; *P* = .06), mirroring our overall findings. No difference between treatments was seen for CPP (unadjusted β, 0.41; 95% CI, −2.77 to 3.58; *P* = .80; adjusted β, −0.21; 95% CI, −4.21 to 3.80; *P* = .92) (eTable 11 in Supplement 1). Because the osmolar load of boluses was 1.88 times greater for 3% HTS, we assessed maximum serum osmolarity observed. Surprisingly, the maximum serum osmolarity observed was lowest in patients in the HTS-only group (mean [SD], 312.90 [39.76] mOsm/kg vs 331.66 [23.82] mOsm/kg in the mannitol-only group and 330.14 [23.73] mOsm/kg in the HTS and mannitol group) (eTable 12 in Supplement 1).

## Discussion

The ADAPT trial was conceived based on the recognition of 3 factors: (1) evidence-based treatment recommendations in the current guidelines reflected a low level of evidence, (2) failure of randomized clinical trials (RCTs) in pediatric sTBI (and parallel failures of RCTs in adults) suggested the need for an alternative approach, and (3) practice variability represented a key contributor to treatment failure in RCTs yet a potential opportunity to compare strategies that are in clinical practice. The ADAPT cohort mimics patients observed in RCTs, such as male preponderance, age, and GCS score.^19^ However, ADAPT is unique in its inclusion of abusive head trauma and penetrating TBI (24% of the overall cohort). Thus, ADAPT more fully reflects patients receiving guidelines-based ICP-directed care than RCTs.

### Practice Variability in Hyperosmolar Therapy

Given our study’s inclusion of clinical sites in 8 countries, hyperosmolar therapy was commonly used for children with sTBI, with 787 children receiving hyperosmolar therapy during the ICP-directed phase of care and 883 receiving such therapy when the prehospital and resuscitation phases of care were included. This finding is reassuring given that hyperosmolar therapy is the only ICP-directed therapy supported by level 2 evidence in the guidelines.^9,10^ The limited used of mannitol vs HTS during the ICP-directed therapy phase also represents a progressive increase in the use of HTS from a previous report.^20^ We observed marked practice variability related to hyperosmolar therapies. In 2012, the guidelines provided level 2 evidence supporting the use of an infusion of 3% HTS at a dose of 6.5 to 10 mL/kg for 2 hours and level 3 evidence for use of a continuous infusion of HTS at an hourly dose of 0.1 to 1.0 mL/kg administered to maintain ICP less than 20 mm Hg.^9^ During our enrollment period, additional support for bolus administration of 3% HTS was published by Shein et al,^7^ who reported a benefit of a dose between 2 and 5 mL/kg given over 10 to 20 minutes on ICP and CPP in 16 patients. Thus, the current guidelines recommend administration of a 3% HTS bolus with level 2 evidence.^10^ Although some practice variability in the use of HTS was anticipated, the use of 27 different concentrations administered as infusions or boluses was surprising. This practice variability was observed at sites that provide high-level care for patients with sTBI (routine placement of an ICP monitor or other maneuvers) and mirrors reports on other facets of care.^21^ Nevertheless, it directed us to focus this analysis specifically on the use of 3% HTS vs mannitol boluses.

### Current Recommendations for Hyperosmolar Therapy 

Current guidelines include a level 2 recommendation for a 3% HTS bolus at doses between 2 and 5 mL/kg for patients with intracranial hypertension, defined as an ICP greater than 20 mm Hg. In a subsequent literature search, we identified 8 additional studies on HTS. A single-center study^22^ compared mannitol and HTS in 30 patients in India and reported a 5– to 7–mm Hg decrease in ICP and a 6–mm Hg increase in CPP that did not differ between groups. In contrast, a retrospective study^23^ of 16 children did not detect a significant reduction or increase in CPP after administration of HTS or mannitol. A retrospective study^24^ compared 3%, 6%, and 12% HTS in 43 children with sTBI and, surprisingly, found a concentration-dependent reduction in ICP of 4.5, 6.6, and 10.5 mm Hg, respectively, despite using equimolar doses of the medications. Three studies^25,26,27^ addressed prehospital or emergency department use. Finally, 2 systematic reviews^28,29^ concluded that there was a paucity of high-quality data, making it difficult to draw conclusions.

We analyzed nearly 2500 boluses of hyperosmolar medications on ICP and CPP in 518 children with sTBI during their routine care. This number represents a substantial increase in sample size compared with the previous studies^9,10^ that informed the guidelines and those outlined earlier. Because both medications were administered relatively frequently and because our baseline data collection to control for confounders was robust, our ability to draw valid conclusions from our analyses was enhanced. On average, routine use of 3% HTS by clinicians delivered nearly twice the osmolar load compared with routine use of mannitol—a fact that may not be recognized at the bedside because calculation of osmolar load is not routine. Nevertheless, the peak serum osmolarity was not higher in the HTS group. Overall, HTS but not mannitol was associated with a significant, albeit relatively modest, reduction in ICP during the initial hour of treatment, whereas both treatments were associated with a modest increase in CPP. The association of these therapies with ICP and CPP could be important. Reduction of ICP can prevent brain deformation and/or herniation, whereas increases in CPP can augment cerebral blood flow. Direct comparison of the therapies also supported superiority of HTS to mannitol on ICP in an unadjusted model, but the difference was not seen after adjustment for confounders, which included osmolar equivalence. The greater osmolar load used in clinical situations among the general population may explain some of the observed success of HTS vs mannitol, but differential changes in blood rheology, cell volume, cardiac output, diuresis, and inflammation may affect the observed responses.

An unanticipated finding is that the overall association of hyperosmolar therapies with ICP and CPP appeared more modest than in previous studies^7,22^ in which typical reductions in ICP after HTS or mannitol were approximately 5 mm Hg. Several factors may have contributed. Although we compared ICP and CPP in the hour before and after the bolus, the values were only collected hourly. Thus, we likely did not capture the most severe derangement that prompted the clinical response, leading to an underestimate of the treatment outcome. In addition, previous studies^3,7,24^ have limited analyses to instances when ICP was generally greater than 20 mm Hg, which we observed for only 21.6% of boluses. It is possible that the care encompassed by ADAPT includes bolus hyperosmolar administration on a fixed schedule to prevent intracranial hypertension, again leading to a diminishment of the observed treatment outcome. When we examined the outcome of bolus administration of either agent in patients with intracranial hypertension (ICP >20, >25, or >30 mm Hg), treatment outcomes consistent with prior studies^3,7,24^ were observed. The use of decompressive craniectomy and cerebrospinal fluid diversion, both of which might affect the hyperosmolar therapies, was common. Lastly, the timing of the use of hyperosmolar therapy could also influence the magnitude of its effect.^16^ However, when we examined the first dose of hyperosmolar therapy, the associations with ICP and CPP mirrored those of the overall cohort.

### Limitations

This study has some limitations. The marked practice variability in the use of hyperosmolar therapy (infusion, bolus, combination therapy, and concentrations) was so great that it was necessary to focus only on bolus administration of medications and only 3% HTS, limiting our ability to compare the various preparations used. We focused on hyperosmolar therapies during a 1-hour epoch, and although well supported, this approach may not fully capture acute vascular and more sustained parenchymal actions. Two centers contributed approximately 40% of the data on mannitol boluses, which could introduce bias. Finally, although ADAPT included valuable data on patients with abusive head trauma and penetrating TBI, these factors might alter the association between hyperosmolar therapy and ICP and CPP. Additional studies of these subgroups are warranted.

## Conclusions

In a large observational comparative effectiveness research study of children with sTBI, marked practice variability was observed in the use of hyperosmolar therapy. When assessing nearly 2500 boluses of 3% HTS or mannitol, overall, bolus administration of 3% HTS was associated with reduced ICP and increased CPP, whereas mannitol only was associated with increased CPP. After adjustment for confounders, the overall associations of both therapies with ICP and CPP were modest and not different. During increased ICP periods, greater outcomes from hyperosmolar therapy were observed, and 3% HTS was associated with greater improvements than mannitol.

## Notes

Supplement 1. **eTable 1.** Definitions of Data Elements **eTable 2.** Concentrations (%) of Hypertonic Saline Administered in Patients During the First 7 Days in ICU (749 Patients Received 31402 Hourly Recordings of Hypertonic Saline Therapy) **eTable 3.** Concentrations (%) of All Administered Hypertonic Saline Boluses (N Total = 2174) **eTable 4.** All Baseline Characteristics of Patients Included in the Analysis **eTable 5.** Sites of Included Patients Who Received 3% Hypertonic Saline or Mannitol Boluses During the First 7 Days in ICU (N Total = 518) **eTable 6.** Counts of 3% Hypertonic Saline Boluses Administered in Patients (N Total = 413) **eTable 7.** Counts of Mannitol Boluses Administered in Patients (N Total = 179) **eTable 8.** Days When Included 3% Hypertonic Saline and Mannitol Boluses Were Administered **eTable 9.** 3% Hypertonic Saline and Mannitol Boluses Stratified by the ICP Level Recorded in the Hour Before the Dose **eTable 10.** Unadjusted and Adjusted Associations of 3% Hypertonic Saline Versus Mannitol With the Change of CPP (mm Hg) After a Bolus (Hour After – Hour Before) Stratified by the ICP (mm Hg) Level Recorded in the Hour Before the Dose **eTable 11.** Unadjusted and Adjusted Associations of 3% Hypertonic Saline Versus Mannitol With the Change of ICP (mm Hg) and CPP (mm Hg) After the First Dose **eTable 12.** Maximum Serum Osmolarity During First 7 Days in ICU of Included Patients **eFigure 1.** Dosing (ml/kg) of Included 3% Hypertonic Saline Boluses **eFigure 2.** Dosing (gram/kg) of Included Mannitol Boluses

Supplement 2. **Nonauthor Collaborators.** The ADAPT Investigators
