# Supplementary material for: Comparison of Intracranial Pressure Measurements Before and After Hypertonic Saline or Mannitol Treatment in Children With Severe Traumatic Brain Injury
Source: JAMA Netw Open. 2022 Mar 10;5(3):e220891. doi: 10.1001/jamanetworkopen.2022.0891 (PMC8914575; doi:10.1001/jamanetworkopen.2022.0891)
Supplement: Supplement 1. — eTable 1. Definitions of Data Elements eTable 2. Concentrations (%) of Hypertonic Saline Administered in Patients During the First 7 Days in ICU (749 Patients Received 31402 Hourly Recordings of Hypertonic Saline Therapy) eTable 3. Concentrations (%) of All Administered Hypertonic Saline Boluses (N Total = 2174) eTable 4. All Baseline Characteristics of Patients Included in the Analysis eTable 5. Sites of Included Patients Who Received 3% Hypertonic Saline or Mannitol Boluses During the First 7 Days in ICU (N Total = 518) eTable 6. Counts of 3% Hypertonic Saline Boluses Administered in Patients (N Total = 413) eTable 7. Counts of Mannitol Boluses Administered in Patients (N Total = 179) eTable 8. Days When Included 3% Hypertonic Saline and Mannitol Boluses Were Administered eTable 9. 3% Hypertonic Saline and Mannitol Boluses Stratified by the ICP Level Recorded in the Hour Before the Dose eTable 10. Unadjusted and Adjusted Associations of 3% Hypertonic Saline Versus Mannitol With the Change of CPP (mm Hg) After a Bolus (Hour After – Hour Before) Stratified by the ICP (mm Hg) Level Recorded in the Hour Before the Dose eTable 11. Unadjusted and Adjusted Associations of 3% Hypertonic Saline Versus Mannitol With the Change of ICP (mm Hg) and CPP (mm Hg) After the First Dose eTable 12. Maximum Serum Osmolarity During First 7 Days in ICU of Included Patients eFigure 1. Dosing (ml/kg) of Included 3% Hypertonic Saline Boluses eFigure 2. Dosing (gram/kg) of Included Mannitol Boluses [file jamanetwopen-e220891-s001.pdf]

Supplementary Online Content

Kochanek PM, Adelson PD, Rosario BL, et al; ADAPT Investigators. Comparison of intracranial pressure measurements before and after hypertonic saline or mannitol treatment in children with severe traumatic brain injury. *JAMA Netw Open*. 2022;5(3):e220891. doi:10.1001/jamanetworkopen.2022.0891

eTable 1. Definitions of Data Elements

eTable 2. Concentrations (%) of Hypertonic Saline Administered in Patients During the First 7 Days in ICU (749 Patients Received 31402 Hourly Recordings of Hypertonic Saline Therapy)

eTable 3. Concentrations (%) of All Administered Hypertonic Saline Boluses (N Total =2174)

eTable 4. All Baseline Characteristics of Patients Included in the Analysis

eTable 5. Sites of Included Patients Who Received 3% Hypertonic Saline or Mannitol Boluses During the First 7 Days in ICU (N Total = 518)

eTable 6. Counts of 3% Hypertonic Saline Boluses Administered in Patients (N Total = 413)

eTable 7. Counts of Mannitol Boluses Administered in Patients (N Total = 179)

eTable 8. Days When Included 3% Hypertonic Saline and Mannitol Boluses Were Administered

eTable 9. 3% Hypertonic Saline and Mannitol Boluses Stratified by the ICP Level Recorded in the Hour Before the Dose

eTable 10. Unadjusted and Adjusted Associations of 3% Hypertonic Saline Versus Mannitol With the Change of CPP (mm Hg) After a Bolus (Hour After – Hour Before) Stratified by the ICP (mm Hg) Level Recorded in the Hour Before the Dose

eTable 11. Unadjusted and Adjusted Associations of 3% Hypertonic Saline Versus Mannitol With the Change of ICP (mm Hg) and CPP (mm Hg) After the First Dose

eTable 12. Maximum Serum Osmolarity During First 7 Days in ICU of Included Patients

eFigure 1. Dosing (ml/kg) of Included 3% Hypertonic Saline Boluses

eFigure 2. Dosing (gram/kg) of Included Mannitol Boluses

This supplementary material has been provided by the authors to give readers additional information about their work.

© 2022 Kochanek PM et al. *JAMA Network Open.*

eTable 1. Definitions of Data Elements

Age	Chronological age at the time of intracranial pressure monitor insertion.
Glasgow Coma Scale (GCS) score	GCS score obtained at the study hospital that placed the intracranial pressure monitor and caused the monitor to be placed (qualifying examination).
Total	GCS Total score on a scale of 3-15
Eye	GCS Eye score on a scale of 1-4
Motor	GCS Motor score on a scale of 1-6
Verbal	GCS Verbal score on a scale of 1-5
Sex	Sex documented within the medical record.
Race	Race of patient within medical record. White, Black and Other were combined from this analysis.
Latino	Ethnicity of patient within medical record. If applicable, it indicates how the patient most closely identifies themselves as “Hispanic or Latino” or “Not Hispanic or Latino”.
Cause of TBI	Cause of traumatic brain injury recorded from a list of options from the Common Data Elements. Motor vehicle collision, Fall, Homicide/assault, and Other were combined from this analysis.
Type of TBI	Type of traumatic brain injury recorded from a list of options from the Common Data Elements. Open and Closed were combined from this analysis.
Mechanism of TBI	Mechanism of traumatic brain injury recorded from a list of options from the Common Data Elements. Acceleration/deceleration, Direct impact/Fall and Penetrating were combined from this analysis.
Likelihood of injury due to abuse[13]	Instructions were made to determine the likelihood of injury due to abuse. Specifically, subjects who had documentation in the medical record of a confirmed diagnosis of child abuse or had clinical notes from the treating physicians that stated that the diagnosis of child abuse was being strongly considered in their differential diagnosis were categorized as “child abuse” cases.
Likelihood of intentional injury	Instructions were made to determine the likelihood that injury was intentional
Likelihood of self-inflicted injury	Instructions were made to determine the likelihood that injury was self-inflicted
Likelihood of injury under the influence of alcohol and/or drugs	Instructions were made to determine the likelihood that the subject was under the influence of alcohol/drugs at the time of the injury.
Transported to study hospital from	Instructions were made to determine the mode of transportation to the study hospital.
Calendar year of enrollment	Calendar year when patient was enrolled
Fixed pupils	Site personnel were instructed to determine if both pupils were fixed, one pupil was fixed or neither pupil was fixed.
Abbreviated Injury Score (AIS)	AIS scores generated from reviewing the medical records of study children in accordance with the AIS manual (distributed to all study sites).
Injury Severity Score (ISS)	ISS generated from reviewing the medical records of study children in accordance with the manual.
Pediatric Risk of Mortality (PRISM) III	PRISM III measures were obtained in accordance with published norms. Importantly, values were obtained within the first 12 h of admission to the

© 2022 Kochanek PM et al. *JAMA Network Open.*

	study hospital. A Pediatric Risk of Mortality III score was calculated for each patient.
Pre-hospital or resuscitation events: Events within this category are intended to have occurred prior to arrival at the study hospital or from the time of arrival to the study hospital to the placement of the intracranial pressure monitor.	
Apnea	Apnea is defined as a cessation of breathing for 20 sec or longer or a shorter respiratory pause that is associated with bradycardia, cyanosis, pallor, and/or marked hypotonia.
Aspiration	Aspiration is defined as the drawing of a foreign substance, such as gastric contents, into the respiratory tract during inhalation.
Cardiac arrest	Cardiac arrest is defined as the cessation of heart function sufficiently severe to require chest compressions.
Hypotension	Hypotension is defined based on the systolic blood pressure (SBP) as follows: neonates (0–28 days of age), SBP <60mm Hg; infants from 1 month to 12 months, SBP <70mm Hg; children >1 year to 10 years, SBP <70 + (2*age in years); children older than 10 years, SBP <90mm Hg.
Hypoxia	Hypoxia is defined as oxygen saturation <90% for 30 min.
Seizure	Seizure is defined as a seizure diagnosed by the care team in the medical record or diagnosed by electroencephalography by a neurologist.
Hyperthermia	Hyperthermia is defined as a rectal temperature >38°C for at least 1 recording.
Hypothermia	Hypothermia is defined as rectal temperature <35.5°C for at least 1 recording.
Hyperventilation	Hyperventilation is defined as an arterial carbon dioxide concentration or end-tidal CO2 < 30mm Hg for at least 1 recording.
Anticonvulsant medication	Site personnel were instructed to check all that apply for anticonvulsants including phenytoin, leviteracetam, phenobarbital, oxcarbamezepine, primidone, topiramate, carbamazepine, valproic acid, or other anticonvulsant. For this analysis, a single agent was required to answer ‘‘yes’’ in the analysis.
Hypertonic saline medication	Indicated ‘‘yes’’ if a hypertonic saline solution was administered.
Mannitol medication	Indicated ‘‘yes’’ if mannitol was administered.
Pentobarbital medication	Indicated ‘‘yes’’ if a barbiturate was administered.
Fluids in, ml/kg/hr	Total amount of all fluids administered was calculated.
Fluids out, ml/kg/hr	Total amount of all fluid output was calculated.
Site	Site where patient was enrolled

© 2022 Kochanek PM et al. *JAMA Network Open.*

eTable 2. Concentrations (%) of Hypertonic Saline Administered in Patients During the First 7 Days in ICU (749 Patients Received 31402 Hourly Recordings of Hypertonic Saline Therapy)

Concentration	N (%)
1	3 (0.01)
1.2	13 (0.04)
1.3	30 (0.10)
1.5	22 (0.07)
1.7	30 (0.10)
1.8	118 (0.38)
1.9	14 (0.04)
2	1003 (3.19)
2.2	20 (0.06)
2.3	6 (0.02)
2.5	25 (0.08)
2.6	20 (0.06)
2.7	305 (0.97)
2.8	11 (0.04)
2.9	8 (0.03)
3	26343 (83.89)
5	235 (0.75)
6	1511 (4.81)
6.4	21 (0.07)
7.5	52 (0.17)
10	470 (1.50)
12	809 (2.58)
13.2	20 (0.06)
18	130 (0.41)
20	70 (0.22)
23	6 (0.02)
23.4	107 (0.34)

© 2022 Kochanek PM et al. *JAMA Network Open.*

eTable 3. Concentrations (%) of All Administered Hypertonic Saline Boluses (N Total =2174)

Concentration	N (%)
1.8	1 (0.05)
2	31 (1.43)
2.7	111 (5.11)
3	1642 (75.53)
5	213 (9.80)
6	5 (0.23)
6.4	18 (0.83)
12	3 (0.14)
13.2	1 (0.05)
18	125 (5.75)
20	3 (0.14)
23	2 (0.09)
23.4	19 (0.87)

© 2022 Kochanek PM et al. *JAMA Network Open.*

eTable 4. All Baseline Characteristics of Patients Included in the Analysis

Characteristics, n (%) or Mean ± SD	Total n=518
**Age**	7.6 ± 5.4
**GCS Total**	5.2 ± 1.8
**GCS Eye**	1.2 ± 0.5
**GCS Motor**	2.9 ± 1.7
**GCS Verbal**	1.1 ± 0.4
**Sex**	
Female	182 (35.1)
Male	336 (64.9)
**Primary race**	
White	274 (52.9)
Black	115 (22.2)
Other	100 (19.3)
Unknown/Withheld	29 (5.6)
**Latino**	
N/A	188 (36.8)
No	272 (53.2)
Yes	51 (10.0)
**Cause of TBI**	
Motor vehicle	289 (55.8)
Fall	103 (19.9)
Homicide/Assault	77 (14.9)
Other	49 (9.5)
**Type of TBI**	
Open	51 (9.8)
Closed	467 (90.2)
**Mechanism of TBI**	
Acceleration/Deceleration	49 (9.6)
Direct impact/Fall	429 (83.8)
Penetrating	34 (6.6)
**Likelihood of injury due to abuse**	
No concern	426 (82.2)
Possible	26 (5.0)
Probable	34 (6.6)
Definite	32 (6.2)
**Likelihood of intentional injury**	
No concern	405 (78.2)
Possible	43 (8.3)
Probable	25 (4.8)
Definite	45 (8.7)
**Likelihood of self-inflicted injury**	
No concern	499 (96.3)
Possible/Probable/Definite	19 (3.7)

© 2022 Kochanek PM et al. *JAMA Network Open.*

**Likelihood of injury under the influence of alcohol and/or drugs**	
None	483 (96.8)
Suspected/Confirmed	16 (3.2)
**Transported to study hospital from**	
Scene of accident/injury	249 (48.1)
Other hospital	244 (47.1)
Home	25 (4.8)
**Calendar year of enrollment**	
2014	103 (19.9)
2015	226 (43.6)
2016	189 (36.5)
**Fixed pupil(s)**	
Both	117 (22.6)
Either	51 (9.8)
Neither	310 (59.8)
Unable to assess/Unknown	40 (7.7)
**AIS Head**	4.2 ± 0.9
**AIS Face**	1.0 ± 1.0
**AIS Neck**	0.2 ± 0.7
**AIS Thorax**	0.9 ± 1.4
**AIS Abdomen**	0.5 ± 1.1
**AIS Spine**	0.3 ± 0.9
**AIS Upper Extremity**	0.4 ± 0.8
**AIS Lower Extremity**	0.6 ± 1.1
**AIS External**	0.4 ± 0.7
**ISS**	26.3 ± 11.6
**PRISM III Score**	17.0 ± 9.1
**Pre-hospital or resuscitation events**	
**Apnea**	
No/Unknown	401 (77.4)
Suspected	63 (12.2)
Yes	54 (10.4)
**Aspiration**	
No/Unknown	439 (84.7)
Suspected	56 (10.8)
Yes	23 (4.4)
**Cardiac arrest**	
No	475 (91.7)
Yes	43 (8.3)
**Hypotension**	
No	368 (71.0)
Yes	150 (29.0)
**Hypoxia**	
No	473 (91.3)
Yes	45 (8.7)
**Seizure**	
No	413 (79.7)

© 2022 Kochanek PM et al. *JAMA Network Open.*

Yes	105 (20.3)
**Hyperthermia**	
No	473 (91.3)
Yes	45 (8.7)
**Hypothermia**	
No	415 (80.1)
Yes	103 (19.9)
**Hyperventilation**	
No	433 (83.6)
Yes	85 (16.4)
**Anticonvulsant medication**	
No	267 (51.5)
Yes	251 (48.5)
**Hypertonic saline medication**	
No	326 (62.9)
Yes	192 (37.1)
**Mannitol medication**	
No	359 (69.3)
Yes	159 (30.7)
**Pentobarbital medication**	
No	504 (97.3)
Yes	14 (2.7)
**Fluids in, ml/kg/hr**	11.0 ± 16.0
**Fluids out, ml/kg/hr**	4.7 ± 9.8
**CT findings**	
**Epidural hematoma**	
Absent	461 (90.9)
Present	46 (9.1)
**Subdural hematoma**	
Absent	162 (31.8)
Present	347 (68.2)
**Intracerebral hemorrhage**	
Absent	210 (41.3)
Present	298 (58.7)
**Intraventricular hemorrhage**	
Absent	390 (76.8)
Present	118 (23.2)
**Subarachnoid hemorrhage**	
Absent	255 (50.2)
Present	253 (49.8)
**Midline shift supratentorial**	
Absent	310 (61.0)
Present	198 (39.0)
**Cisternal compression**	
Absent	294 (57.9)
Present	214 (42.1)
**Diffuse axonal injury**	

© 2022 Kochanek PM et al. *JAMA Network Open.*

Absent	367 (72.2)
Present	141 (27.8)
**Contusion**	
Absent	257 (50.6)
Present	251 (49.4)
**Brain swelling**	
Absent	191 (37.6)
Present	317 (62.4)
**Ischemia or infarction or hypoxic-ischemic injury**	
Absent	437 (85.9)
Present	72 (14.1)
**Site name**	
Pittsburgh	12 (2.3)
Atlanta	42 (8.1)
Detroit	23 (4.4)
DC Children's	14 (2.7)
Boston	4 (0.8)
Johns Hopkins	9 (1.7)
Charlotte	12 (2.3)
MGH	4 (0.8)
Miami	1 (0.2)
Columbus	7 (1.4)
Phoenix	27 (5.2)
Hershey	7 (1.4)
Houston	8 (1.5)
UC Davis	17 (3.3)
UAB	10 (1.9)
UCLA	3 (0.6)
San Diego	12 (2.3)
Cincinnati	19 (3.7)
CHOP	9 (1.7)
USC	3 (0.6)
Memphis	15 (2.9)
UTSW	36 (6.9)
Seattle	11 (2.1)
St Louis	13 (2.5)
Wisconsin	3 (0.6)
Birmingham	15 (2.9)
Barcelona	2 (0.4)
Newcastle	1 (0.2)
Manchester	1 (0.2)
Liverpool	4 (0.8)
Cambridge	2 (0.4)
Leeds	1 (0.2)
Southampton	3 (0.6)
Auckland	8 (1.5)
New Delhi	62 (12.0)

© 2022 Kochanek PM et al. *JAMA Network Open.*

Melbourne	22 (4.2)
Perth	6 (1.2)
Brisbane	10 (1.9)
VCU	8 (1.5)
Iowa	11 (2.1)
Omaha	4 (0.8)
Denver	23 (4.4)
Utah	7 (1.4)
Vanderbilt	7 (1.4)

© 2022 Kochanek PM et al. *JAMA Network Open.*

eTable 5. Sites of Included Patients Who Received 3% Hypertonic Saline or Mannitol Boluses During the First 7 Days in ICU (N Total = 518)

Site name	N (%)
Pittsburgh	12 (2.3)
Atlanta	42 (8.1)
Detroit	23 (4.4)
DC Children’s	14 (2.7)
Boston Children’s	4 (0.8)
Johns Hopkins	9 (1.7)
Charlotte	12 (2.3)
MGH	4 (0.8)
Miami	1 (0.2)
Columbus	7 (1.4)
Phoenix	27 (5.2)
Hershey	7 (1.4)
Houston	8 (1.5)
UC Davis	17 (3.3)
UAB	10 (1.9)
UCLA	3 (0.6)
San Diego	12 (2.3)
Cincinnati	19 (3.7)
CHOP	9 (1.7)
USC	3 (0.6)
Memphis	15 (2.9)
UTSW	36 (6.9)
Seattle	11 (2.1)
St Louis	13 (2.5)
Wisconsin	3 (0.6)
Birmingham	15 (2.9)
Barcelona	2 (0.4)
Newcastle	1 (0.2)
Manchester	1 (0.2)
Liverpool	4 (0.8)
Cambridge	2 (0.4)
Leeds	1 (0.2)
Southampton	3 (0.6)
Auckland	8 (1.5)
New Delhi	62 (12.0)
Melbourne	22 (4.2)
Perth	6 (1.2)
Brisbane	10 (1.9)
VCU	8 (1.5)
Iowa	11 (2.1)
Omaha	4 (0.8)
Denver	23 (4.4)
Utah	7 (1.4)
Vanderbilt	7 (1.4)

© 2022 Kochanek PM et al. *JAMA Network Open.*

eTable 6. Counts of 3% Hypertonic Saline Boluses Administered in Patients (N Total = 413)

Count	N (%)
1	145 (35.1)
2	66 (16.0)
3	53 (12.8)
4	34 (8.2)
5	26 (6.3)
6	16 (3.9)
7	12 (2.9)
8	13 (3.2)
9	16 (3.9)
10	11 (2.7)
11	1 (0.2)
12	2 (0.5)
13	2 (0.5)
14	1 (0.2)
15	1 (0.2)
16	1 (0.2)
17	3 (0.7)
18	1 (0.2)
19	3 (0.7)
21	1 (0.2)
22	2 (0.5)
23	2 (0.5)
25	1 (0.2)

© 2022 Kochanek PM et al. *JAMA Network Open.*

eTable 7. Counts of Mannitol Boluses Administered in Patients (N Total = 179)

Count	N (%)
1	74 (41.3)
2	22 (12.3)
3	11 (6.2)
4	5 (2.8)
5	7 (3.9)
6	5 (2.8)
7	5 (2.8)
8	5 (2.8)
9	11 (6.2)
10	8 (4.5)
11	3 (1.7)
12	6 (3.4)
13	3 (1.7)
14	2 (1.1)
15	3 (1.7)
16	2 (1.1)
18	1 (0.6)
19	1 (0.6)
20	1 (0.6)
21	2 (1.1)
23	1 (0.6)
25	1 (0.6)

© 2022 Kochanek PM et al. *JAMA Network Open.*

eTable 8. Days When Included 3% Hypertonic Saline and Mannitol Boluses Were Administered

PICU Day Number - n (%N)	Total N=2494	3% hypertonic saline N=1608	Mannitol N=886	P
1	753 (30.2)	530 (33.0)	223 (25.2)	*0.02
2	516 (20.7)	321 (20.0)	195 (22.0)	
3	374 (15.0)	220 (13.7)	154 (17.4)	
4	274 (11.0)	147 (9.1)	127 (14.3)	
5	212 (8.5)	125 (7.8)	87 (9.8)	
6	184 (7.4)	130 (8.1)	54 (6.1)	
7	181 (7.3)	135 (8.4)	46 (5.2)	

* A univariate generalized linear mixed model with the hyperosmolar therapy as the dependent variable, the PICU Day as an independent variable and the patient level as the random effect was used for the statistical test.

© 2022 Kochanek PM et al. *JAMA Network Open.*

eTable 9. 3% Hypertonic Saline and Mannitol Boluses Stratified by the ICP Level Recorded in the Hour Before the Dose

ICP (mmHg) hour before - n (%N)	Total N=1972	3% hypertonic saline N=1326	Mannitol N=646
ICP hour before ≤ 20	1545 (78.4)	1027 (77.4)	518 (80.2)
ICP hour before > 20	427 (21.6)	299 (22.6)	128 (19.8)

© 2022 Kochanek PM et al. *JAMA Network Open.*

eTable 10. Unadjusted and Adjusted Associations of 3% Hypertonic Saline Versus Mannitol With the Change of CPP (mm Hg) After a Bolus (Hour After – Hour Before) Stratified by the ICP (mm Hg) Level Recorded in the Hour Before the Dose

CPP (hour after – hour before)	Unadjusted β [95% CI]	P	Adjusted β [95% CI]	P
Stratum: ICP hour before ≤ 20	-0.42 [-1.69 – 0.86]	0.52	-0.92 [-2.40 – 0.56]	0.22
Stratum: ICP hour before > 20	1.18 [-1.32 – 3.69]	0.35	-0.06 [-3.00 – 2.88]	0.97
Stratum: ICP hour before ≤ 25	-0.16 [-1.38 – 1.05]	0.79	-0.75 [-2.15 – 0.65]	0.29
Stratum: ICP hour before > 25	2.04 [-1.31 – 5.39]	0.23	0.58 [-3.42 – 4.58]	0.78
Stratum: ICP hour before ≤ 30	0.15 [-1.05 – 1.35]	0.81	-0.52 [-1.90 – 0.87]	0.46
Stratum: ICP hour before > 30	-0.20 [-4.40 – 4.01]	0.93	-2.20 [-7.35 – 2.94]	0.40

All adjusted models were adjusted for GCS total, GCS motor, sex, AIS thorax, AIS abdomen, AIS upper extremity, hyperventilation in pre-hospital/resuscitation, and anticonvulsant medication in pre-hospital/resuscitation.

© 2022 Kochanek PM et al. *JAMA Network Open.*

eTable 11. Unadjusted and Adjusted Associations of 3% Hypertonic Saline Versus Mannitol With the Change of ICP (mm Hg) and CPP (mm Hg) After the First Dose

Outcomes	Unadjusted β [95% CI]	P	Adjusted β [95% CI] ¶	P
ICP (hour after – hour before)	-1.99 [-3.82 - -0.16]	0.03	-2.74 [-5.61 – 0.13]	0.06
CPP (hour after – hour before)	0.41 [-2.77 – 3.58]	0.80	-0.21 [-4.21 – 3.80]	0.92

¶Propensity score was estimated for each subject by modeling on all baseline characteristics. Propensity score weighted regression was used to compare the change of ICP and CPP after the administration of a 3% hypertonic saline bolus versus a mannitol bolus

© 2022 Kochanek PM et al. *JAMA Network Open.*

eTable 12. Maximum Serum Osmolarity During First 7 Days in ICU of Included Patients

Maximum serum osmolarity (mOsm/kg)	3% hypertonic saline boluses alone<br>N total =339	Mannitol boluses alone<br>N total =105	Both mannitol and 3% hypertonic saline boluses<br>N total =74	P
N	175	47	49	
Mean ± SD	312.90 ± 39.76	331.66 ± 23.82	330.14 ± 23.73	<0.001
Median (IQR)	314 (299, 328)	332 (316, 344)	328 (312, 351)	

© 2022 Kochanek PM et al. *JAMA Network Open.*

eFigure 1. Dosing (ml/kg) of Included 3% Hypertonic Saline Boluses

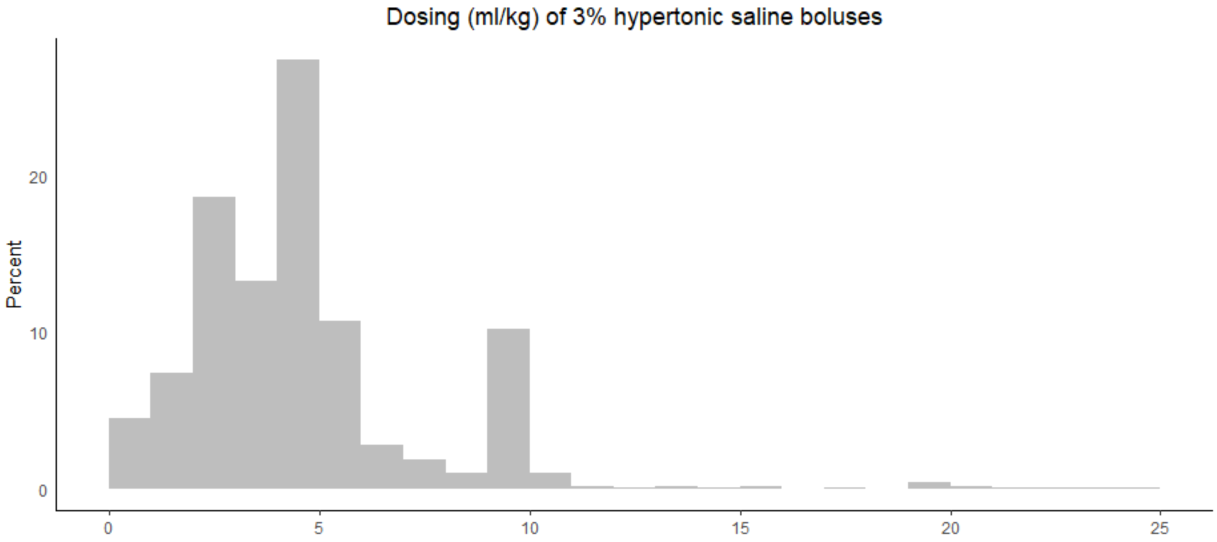

© 2022 Kochanek PM et al. *JAMA Network Open.*

eFigure 2. Dosing (gram/kg) of Included Mannitol Boluses

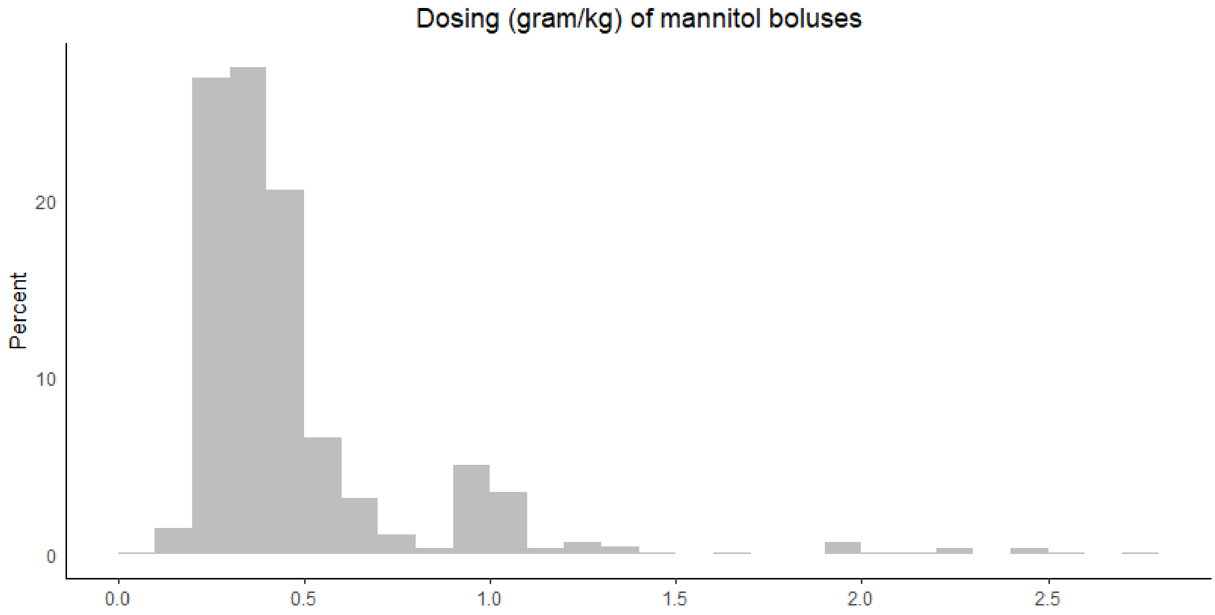

© 2022 Kochanek PM et al. *JAMA Network Open.*
