# Supplementary material for: Comparison of Intracranial Pressure Measurements Before and After Hypertonic Saline or Mannitol Treatment in Children With Severe Traumatic Brain Injury
Source: JAMA Netw Open. 2022 Mar 10;5(3):e220891. doi: 10.1001/jamanetworkopen.2022.0891 (PMC8914575; doi:10.1001/jamanetworkopen.2022.0891)
Supplement: Supplement 2. — Nonauthor Collaborators. The ADAPT Investigators [file jamanetwopen-e220891-s002.pdf]

Supplemental Online Content: Nonauthor Collaborators

*Indicates required information. Only first name, last name, and suffix will appear in PubMed.

*Group Name(s): ADAPT Investigators							
***First Name and Middle Initial(s)**	***Last Name**	***Suffix (eg, Jr, III)**	Academic Degrees	Institution	Location (city, state/province, country)	Role or Contribution, eg, chair, principal investigator	Group (if more than 1 Group listed in the byline) and/or Subgroup (eg, Steering Committee)
Shruti	Agarwal		MD	Addenbrookes Hospital	Cambridge UK	Site investigator	
Sarah	Mahoney		MD	Alder Hey Children's Hospital	Liverpool UK	Site investigator	
Laura	Loftis		MD	Texas Children's Hospital	Houston TX	Site investigator	
Kevin	Morris		MD	University of Birmingham, UK	Birmingham UK	Site investigator	
Lauren	Piper		MD	Levine Children's Hospital	Charlotte NC	Site investigator	
Anthony	Slater		MD	Children's Health Queensland Hospital and Health Service	Brisbane AU	Site investigator	
Karen	Walson		MD	Children's Health Care of Atlanta	Atlanta GA	Site investigator	
Todd	Kilbaugh		MD	Children's Hospital of Philadelphia	Philadelphia PA	Site investigator	
AM Iqbal	O'Meara		MD	Children's Hospital of Richmond	Richmond VA	Site investigator	
Nathan	Dean		MD	Children's National Hospital	Washington, DC	Site investigator	
Jessica	Carpenter		MD	Children's National Hospital	Washington, DC	Site investigator	
Ranjit	Chima		MD	Children's Hospital of Cincinnati	Cincinnati OH	Site investigator	
Brad	Kurowski		MD	Children's Hospital of Cincinnati	Cincinnati OH	Site investigator	
Enno	Wildshut		MD	Erasmus Medical Center	Rotterdam Netherlands	Site investigator	
Naomi	Ketharanathan		MD	Erasmus Medical Center	Rotterdam Netherlands	Site investigator	
Mark	Peters		MD	Great Ormond Street	London UK	Site investigator	
Robert	Tasker		MD	Boston Children's Hospital	Cambridge MA	Site investigator	
Joan	Balcells		MD	Vall d'Hebron Hospital	Barcelona, Spain	Site investigator	
Courtney	Robertson		MD	Johns Hopkins University	Baltimore MD	Site investigator	
Sian	Cooper		MD	Leeds Teaching Hospitals	Leeds UK	Site investigator	

Supplemental Online Content: Nonauthor Collaborators

*Indicates required information. Only first name, last name, and suffix will appear in PubMed.

*First Name and Middle Initial(s)	*Last Name	*Suffix (eg, Jr, III)	Academic Degrees	Institution	Location (city, state/province, country)	Role or Contribution, eg, chair, principal investigator	Group (if more than 1 Group listed in the byline) and/or Subgroup (eg, Steering Committee)
Sarah	Murphy		MD	Massachussetts General Hospital	Boston MA	Site investigator	
Michael	Whalen		MD	Massachussetts General Hospital	Boston, MA	Site investigator	
John	Kuluz		MD	Miami Children's Hospital	Miami FL	Site investigator	
Warwick	Butt		MD	The Royal Children's Hospital of Melbourne	Melbourne AU	Site investigator	
Neal	Thomas		MD	Pennsylvania State University	Hershey PA	Site investigator	
Sandra	Buttram		MD	Phoenix Children's Hospital	Phoenix, AZ	Site investigator	
Simon	Erickson		MD	Perth Children's Hospital	Perth AU	Site investigator	
J. Mahil	Samuel		MD	Royal Manchester Children's Hospital	Manchester UK	Site investigator	
Rachel	Agbeko		MD	Newcastle on Tyne Foundation Trust	Newcastle UK	Site investigator	
Richard	Edwards		MD	Bristol Royal Hospital for Children	Bristol UK	Site investigator	
Iain	Macintosh		MD	University Hospital Southampton	Southampton UK	Site investigator	
Michele	Kong		MD	University of Alabama Birmingham	Birmingham AL	Site investigator	
Joanne	Natale		MD	University of California Davis	Sacramento CA	Site investigator	
Heather	Siefkes		MD	University of California Davis	Sacramento CA	Site investigator	
Christopher	Giza		MD	University of California Los Angeles	Los Angeles CA	Site investigator	
Hari	Thangarajah		MD	University of California San Diego	San Diego CA	Site investigator	
David	Shellington		MD	University of California San Diego	San Diego CA	Site investigator	
Anthony	Figaji		MMED	Red Cross War Memorial Children's Hospital	Capetown South Africa	Site investigator	
Elizabeth	Newell		MD	University of Iowa	Iowa City IA	Site investigator	

Supplemental Online Content: Nonauthor Collaborators

*Indicates required information. Only first name, last name, and suffix will appear in PubMed.

***First Name and Middle Initial(s)**	***Last Name**	***Suffix (eg, Jr, III)**	Academic Degrees	Institution	Location (city, state/province, country)	Role or Contribution, eg, chair, principal investigator	Group (if more than 1 Group listed in the byline) and/or Subgroup (eg, Steering Committee)
Edward	Truemper		MD	University of Nebraska	Omaha NE	Site investigator	
Sidharth	Mahapatra		MD	University of Nebraska	Omaha NE	Site investigator	
Robert	Clark		MD	University of Pittsburgh	Pittsburgh PA	Site investigator	
Alicia	Au		MD	University of Pittsburgh	Pittsburgh PA	Site investigator	
Sue	Beers		PhD	University of Pittsburgh	Pittsburgh PA	Outcomes coordinator	
Christopher	Newth		MD	Children's Hospital of Los Angeles	Los Angeles CA	Site investigator	
Nadeem	Shafi		MD	LeBonheur Children's Hospital	Memphis TN	Site investigator	
Alino Nico	West		PhD	LeBonheur Children's Hospital	Memphis TN	Site investigator	
Darryl	Miles		MD	University of Texas Southwestern	Dallas TX	Site investigator	
Michelle	Schober		MD	University of Utah	Salt Lake City UT	Site investigator	
Truc	Le		MD	University of Vanderbilt	Nashville, TN	Site investigator	
Jerry	Zimmerman		MD	University of Washington	Seattle WA	Site investigator	
Mark	Wainwright		MD	University of Washington	Seattle WA	Site investigator	
Monica	Vavilala		MD	University of Washington	Seattle WA	Site investigator	
Stuart	Friess		MD	Washington University of St. Louis	St. Louis MO	Site investigator	
Jose	Pineda		MD	University of Washington, St. Louis	St. Louis MO	Site investigator	
